# A Challenging Case of Congenital Adrenal Hyperplasia Due to CYP11B1 Deficiency With Uncontrolled Hypertension

**DOI:** 10.1155/crie/1422782

**Published:** 2025-04-11

**Authors:** Pierluigi Mazzeo, Filippo Ceccato, Irene Tizianel, Mattia Barbot

**Affiliations:** ^1^Department of Medicine DIMED, Endocrine Unit, University of Padova, Padua, Italy; ^2^Endocrine Unit, University Hospital of Padova, Padua, Italy

**Keywords:** 11*β*-OHD, Congenital adrenal hyperplasia (CAH), dual-release hydrocortisone, hypertension

## Abstract

Congenital adrenal hyperplasia (CAH) due to 11-beta-hydroxylase deficiency (11*β*-OHD) is the second most common steroidogenesis impairment in European populations, characterized by hypertension, hypokalemia, infertility, hyperandrogenism, and genital ambiguity in females. We present the case of a biological male patient with 11*β*-OHD CAH who developed resistant hypertension, along with massive adrenal enlargement and testicular adrenal rests due to inadequate disease control while on dexamethasone treatment, compounded by drug interactions with his antiepileptic therapy. As the patient was reluctant to switch to a three-times-daily hydrocortisone regimen, he was transitioned to dual-release hydrocortisone, resulting in progressive improvement of most of his symptoms. This case highlights the importance of tailored therapy, particularly in rare diseases.

## 1. Introduction

Congenital adrenal hyperplasia (CAH) is a group of autosomal recessive disorders characterized by impaired cortisol production due to an enzymatic deficiency in steroid synthesis. Among these, 11-beta-hydroxylase deficiency (11*β*-OHD) is the second most common form of CAH in European populations, accounting for ~0.2%–8% of all cases [1, 2]. This condition is caused by reduced or absent activity of 11*β*-hydroxylase (CYP11B1), which is the final step in the synthesis of both aldosterone and cortisol, and the degree of clinical manifestation is related to the residual enzyme activity CYP11B1 [1]. The most common clinical features of 11*β*-OHD are similar to those of 21-hydroxylase deficiency (21-OHD) and include precocious puberty with accelerated skeletal maturation, leading to short stature, ambiguous genitalia in females, and infertility in males [2].

However, a distinguishing feature of 11*β*-OHD is the development of hyporeninemic hypokalemic hypertension, which results from the accumulation of aldosterone precursors, such as 11-deoxycorticosterone (DOC), that possess moderate mineralocorticoid activity [2].

As for 21-OHD, glucocorticoids (GCs) are the mainstay of treatment for 11*β*-OHD. The dual aims of this therapy are to prevent adrenal insufficiency and suppress androgen excess through direct negative feedback on pituitary adrenocorticotropic hormone (ACTH) secretion, while minimizing GC-induced iatrogenic cushingoid features like osteoporosis and a worse cardio-metabolic profile [3]. In fact, it is known that CAH patients have an increased prevalence of cardiovascular (CV) diseases compared to the general population, that is largely attributed to a poor metabolic profile and worsening of other known CV risk factors [3]. The worsening of metabolic profile during GC treatment is first linked to imbalance in GC under- and overtreatment [4]; in addition, also the type of GC used can influence the development of CV risk factors; among conventional GCs, dexamethasone has been associated with a marked deterioration of metabolic profile [5].

Moreover, since those patients required a life-long GC treatment, it should not be underestimated that GCs are both substrates and sometime inducers of cytochromes, so they could have a potential interaction with other concomitant treatments [6]; for this reason, clinicians should be aware of potential drug–drug interactions that complicated the follow-up of these patients.

Here, we describe a case of a patient with CAH due to 11*β*-OHD who developed chronic complications despite continuous GC treatment. The consequences of uncontrolled disease included massive bilateral adrenal enlargement, hypogonadism, testicular adrenal rest tumors (TARTs), and severe hypertension, moreover, were pinpointing the difficulties in the long-term management, especially when treatment resistance and drug interactions occur, focusing on the importance of a tailored therapy.

## 2. Case Presentation

A 26-years-old biological male with a known diagnosis of CAH was referred to our hospital for endocrine assessment due to asthenia, poor biochemical control, and severe drug-resistant arterial hypertension despite being on triple antihypertensive therapy.

He was diagnosed of CAH at the age of two, following early pubarche, and was initially treated with hydrocortisone and fludrocortisone after being misdiagnosed with 21-OHD.

Treatment was adjusted when renin levels were measured, leading to the withdrawal of fludrocortisone. After puberty, since he was reluctant to have three-times-daily administration, his treatment was switched from hydrocortisone to dexamethasone, which he was taking at a dose of 0.25 mg at bedtime. Since his blood pressure began to rise during adolescence, antihypertensive treatment was initiated at age 18. At our first evaluation, he was taking ramipril 10 mg, lercanidipine 20 mg, and canrenone 50 mg.

In terms of his medical history, he also had Hashimoto's disease and developed drug-resistant multifocal partial epilepsy after puberty, requiring continuous adjustments in medication type and dosage. At the time of presentation, his epilepsy was partially controlled with a combination of carbamazepine, valproic acid, and perampanel.

He reported no other family members affected by CAH or any family history of other endocrine diseases.

In October 2014, he was referred to our unit for a complete endocrine assessment, as his blood pressure was no longer under control. He exhibited severe hypertension, particularly with elevated diastolic values (BP 170/110 mmHg), and had a recent history of transient right hemiplegia during a hypertensive crisis, which was attributed to a transient ischemic attack that occurred just 2 months prior.

Physical examination revealed a height of 1.72 m, a weight of 77 kg (BMI 26 kg/m^2^), and normal development of external genitalia. He reported no cigaret smoking, occasional alcohol consumption, and no substance abuse.

A complete endocrine evaluation indicated poor hormone control, with mild hypokalemia (3.3 mmol/L); low plasma renin (<2.0 mUI/L) and aldosterone levels (11 pmol/L); and elevated ACTH (1063 ng/L), androstenedione (111 nmol/L), and 17-hydroxyprogesterone (17-OHP, 45.8 nmol/L) levels (Table 1).

Moreover, a genetic test trough Sanger sequencing and MLPA was conducted to confirm the diagnosis of 11*β*-OHD, revealing a mutation of CYP11B1 (p.W247X) in homozygous form, consistent with the classic form of CAH [7].

The 24-h ambulatory blood pressure monitoring (ABPM) confirmed moderate-severe hypertension throughout the entire day, with median values of 156/94 mmHg. Additionally, we performed a comprehensive screening for complications associated with long-term uncontrolled hypertension; we observed microalbuminuria but no decrease in renal function, and there were no other comorbidities detected during fundoscopic examination, echocardiography, and carotid ultrasound.

**Figure 1:**
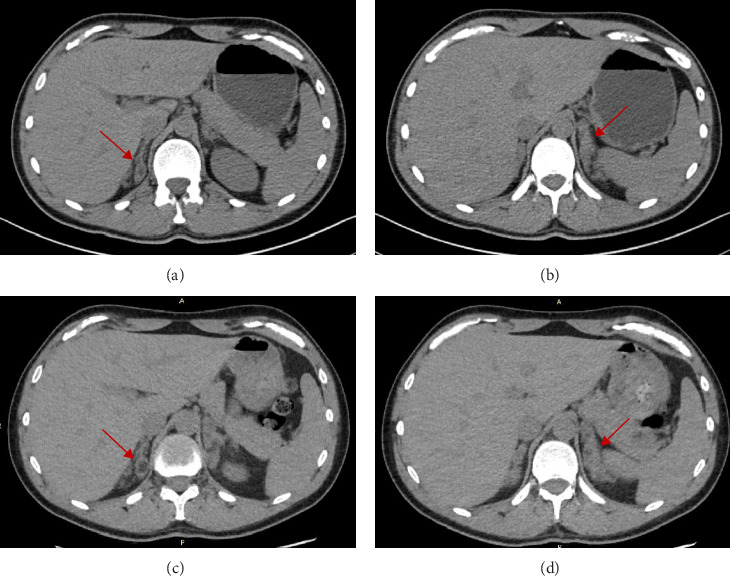
The CT scan at DR-HC start (A and B panels) and at last evaluation, 4 years later (C and D panels). A and C panels highlighted right adrenal enlargement with a hypodense nodule of the medial limb of 13 mm × 18 mm that remained stable during DR-HC treatment; B and D panels showed, instead, a diffuse enlargement of the left adrenal gland due to a long-term uncontrolled disease.

An abdominal CT-scan revealed a globally marked enlargement of both adrenal glands related to poor disease control, with two major nodules measuring 15 mm × 18 mm in the middle leaflet of left adrenal gland and 13 mm × 18 mm in the right adrenal gland (Figure 1).

**Figure 2:**
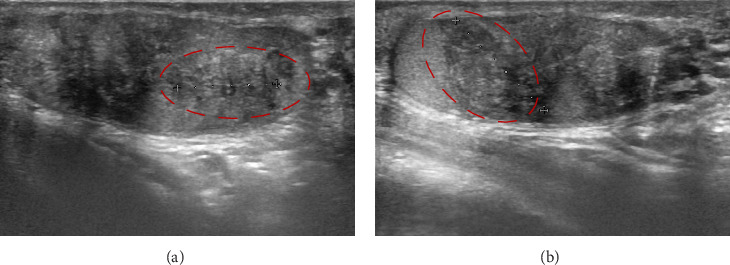
The testicular ultrasound of the right testis showing bilateral TARTs appearing as pseudonodular areas of rounded morphology and ill-defined margins affecting the entire parenchyma of respectively of (A) 17 mm and (B) 12 mm with a predominantly inhomogeneous hypoechoic echostructure with hyperechoic spots. These lesions appeared highly vascularized and are consistent with TARTs, despite sometimes they could be placed into differential diagnosis with other tumor lesions.

Testicular ultrasound detected diffuse inhomogeneous areas throughout the testicular parenchyma and bilateral pseudonodular hypoechoic formations approximately 2 cm in size, with poorly defined margins and increased vascularization, consistent with TARTs (Figure 2).

Regarding gonadal function, total testosterone levels were normal, but low LH and FSH levels and very high androstenedione to total testosterone (A4/T) ratio indicated an excessive adrenal production of androgens (Table 1). Semen analysis revealed azoospermia, likely related to both hypogonadism and mechanical obstruction due to bilateral TARTs.

Initially, we doubled the dexamethasone dosage to 0.50 mg daily at bedtime, but this did not result in any improvement in blood pressure or hormone profile. Consequently, the dose was further increased up to 0.75 mg, yet no positive results were observed, necessitating a progressive increase in antihypertensive therapy. Indeed, the patient was on a triple combination of lisinopril 10 mg, indapamide 2.5 mg, and amlodipine 10 mg in the morning, along with canrenone 200 mg in the morning and 100 mg in the afternoon, and clonidine 300 mg in the evening. Since, the patient was taking carbamazepine and valproic acid, both CYP3A4 inducers, we speculated that there might have been a significant increase in dexamethasone metabolism [8, 9].

Since no changes in the anti-epileptic treatment was feasible and the patient was reluctant to return to a three-time daily administration of hydrocortisone, we decided to switch his GC treatment to dual-release hydrocortisone (DR-HC; Plenadren, Takeda Pharmaceuticals International AG, Dublin, Ireland) at a dosage of 20 mg per day.

A slight improvement in blood pressure control was observed in the first month, with average daily ABP values changing from 155/95 mmHg to 145/90 mmHg.

Six months after the change in treatment, the patient reported an improvement in overall well-being and hormonal control, although it remained suboptimal (17-OHP 16.2 nmol/L, while androstenedione 88.5 nmol/L and A4/T ratio 6.86; Table 1).

Consequently, we increased the dosage of DR-HC to 20 mg in the morning and 5 mg in the early afternoon. Over the following year with self-reported blood pressure and ambulatory evaluations indicating normal levels, antihypertensive therapy was gradually reduced to a daily regimen of canrenone 100 mg and an oral combination of ramipril and amlodipine (5 mg/10 mg). The improvement was confirmed by 24 h ABPM which showed mean diurnal and nocturnal values of 117/73 mmHg and 103/55 mmHg, respectively, with physiological dipping profile, and the good ABP control persists over a 4-years follow-up period.

Furthermore, there was an improvement in testosterone levels (from 12.91 to 17.20 nmol/L, see Table 1) and A4/T ratio (from 6.86 to 4.40) together with a partial restoration of spermatogenesis, transitioning from azoospermia to oligoasthenozoospermia (volume 3 mL, pH 7.2, sperm concentration 10 × 10^6^/mL, total count 30 × 10^6^, viability 44%, motility (A) 0%, (B) 36%, nonprogressive 16%, immobile 48%, and normal morphology 4%) due to a partial reactivation of the hypothalamic–pituitary axis. Since our patient was not seeking fertility, we do not repeat semen analysis over the years.

Lastly, both testicular and adrenal lesions remained stable with DR-HC treatment during the 4-years follow-up period.

## 3. Discussion

This peculiar case report highlights the diagnostic challenge and the wide spectrum of complications that can arise in patients with classic CAH due to 11*β*-OHD and the potential relevance of drug–drug interactions in patients that required a lifelong treatment.

Initially the patient was misdiagnosed because his clinical presentation was consistent with 21-OHD. At that time, hypertension was absent due to the relative resistance of newborns and infants to mineralocorticoids, and unfortunately only 17-OHP was measured, which did not suffice for an accurate diagnosis [4].

First of all, it is important to emphasize that to fully differentiate CAH due to 21-OHD from others enzymatic defects as 11*β*-OHD, clinicians should measure not only 17-OHP and androstenedione levels, but also DOC, 11-deoxycortisol, 17-OH-pregnenolone, and dehydroepiandrosterone levels from blood or urine samples [4]. On the other hand, genetic testing is equally helpful for a precise diagnosis; in our case, we observed mutation of CYP11B1 (p.W247X) in homozygous form; p.W247X is a nonsense pathogenic variant, where the TTG to TAG change in exon four leads to a replace of tryptophan at position 247 by a nonsense codon [7]. This mutation is responsible of classic CAH form when in homozygosis and has a relatively high frequency in populations living in the alpine regions of Tyrol, near where our patient resides, likely due to a founder effect [7].

In adjunction, CAH due to 11*β*-OHD should be suspected also in young patients with hypokalemic hypertension with low renin and aldosteron levels; in fact, sometimes hypokalemic hypertension could be the first sign in patients, especially with nonclassic CAH form that can have mildly elevated to normal 17-OHP levels, and placed in differential diagnosis with other genetic disorders like apparent mineralocorticoid excess syndrome, Liddle's syndrome, and 17 α-hydroxylase deficiency [10, 11].

Apart from the correct diagnosis, in our case the second challenge was to improve hormonal control.

In fact, once correctly diagnosed, the patient was maintained on hydrocortisone and achieved good hormonal control until puberty, after which biochemical control worsened and hydrocortisone was replaced by dexamethasone for patient's will.

It is well established that puberty is associated with significant changes in the endocrine milieu that may lead to alterations in cortisol pharmacokinetic; during puberty it is observed an increase in cortisol clearance and its volume of distribution related to the pubertal rise of GH, IGF-I, and insulin levels that drives modifications mainly in 11*β*-hydroxysteroid dehydrogenase type 1 (11*β*-HSD1) activity and glomerular filtration rate [12]. For those reasons, the management of CAH patients at puberty is often problematic, and requires frequent adjustment of GC doses.

Second, in our patient the onset of a drug-resistant multifocal partial epilepsy greatly complicated the management of his genetic condition due to multiple drug–drug interactions.

Due to the necessity of multiple anti-epileptic drugs (AEDs) assumption to control neurological manifestations, we hypothesized that the worsening of biochemical control was also related to the increased metabolism of dexamethasone; in fact, dexamethasone is a substrate of CYP3A4, and both carbamazepine and valproic acid are known to be CYP3A4 and CYP2B6 inducers [6, 8, 9].

Moreover, dexamethasone itself seems to be a potential CYP3A4 inducer [6] and carbamazepine is also metabolized by this enzyme [8], therefore, the coadministration of dexamethasone with other drugs sensitive for CYP3A4 (both as substrates or perpetrators), resulted in very complex interaction patterns.

Even if we tried to improve hormonal control by increasing dexamethasone doses, at the end we decided to replace this treatment for several reasons; in fact, the AEDs management was really challenging over the years, with frequent necessity to change AEDs type and posology, and most of them had potential interaction with dexamethasone, which could explain the persistent poor disease control in our patient; moreover, due to the delicate balance found in managing his neurological disease, AEDs could not be easily replaced to reduce interactions.

Lastly, apart from the above mention drug interactions, our patient had also an increased CV risk profile despite the young age due to uncontrolled hypertension complicated by a previous cerebrovascular event, and among conventional GCs dexamethasone has been associated with a marked deterioration of the metabolic profile and potential worsening of CV risk profile [5].

Therefore, to minimize drug interactions and reduce metabolic impact, we opted to replace dexamethasone with hydrocortisone; in detail, we chose DR-HC to enhance our patient's poor compliance, since he previously refused to take immediate release hydrocortisone as it required multiple daily administration.

Moreover, DR-HC has been associated with a lower risk of metabolic alterations in patients with adrenal insufficiency due to its ability to better mimic the physiological cortisol rhythm, and similar data are reported also in CAH patients [13, 14].

Notably, hydrocortisone has no direct effects on CYP3A4 activity and even if hydrocortisone is metabolized by this enzyme, it has also additional metabolic pathways, like 11*β*-HSD and 5α-reductase system, which could make it less susceptible to drug-induced hypermetabolism [15].

In fact, despite introducing the same HC equivalent doses, during DR-HC treatment there was a clear improvement of hormonal and ABP control, potentially related to minor treatment resistance and drug interactions.

The other two main issues in this patient were uncontrolled hypertension and hypogonadism with infertility due to both bilateral TARTs development and adrenal androgen excess.

While infertility and TARTs are also common complications in patients with uncontrolled CAH due to 21-OHD, hypertension is typically observed in about two-thirds of patients affected by either 11*β*-OHD and 17*α*-hydroxylase deficiency [2, 3, 16].

Patients with 11*β*-OHD are protected from salt wasting by the excess of mineralocorticoid precursors; however, they can experience adrenal crisis during critical illness due to the complete cortisol deficiency [2]. Fortunately, this was not the case for our patient, who did not have any episodes of adrenal insufficiency throughout his life. DOC is a steroid precursor with moderate mineralocorticoid activity; however, when it accumulates in significant amounts, it can cause sodium retention and volume expansion, leading to severe juvenile hypertension, and hypokalemic hypertension may be the first sign in patients with nonclassic CAH who can present with only mildly elevated to normal 17-OHP levels [2].

Furthermore, one of the primary challenges is maintaining the delicate balance in corticosteroid dosage to reduce ACTH-driven increases in DOC without causing over-replacement, as both conditions can adversely affect metabolic and CV profiles, as well documented in patients with 21-OHD [3].

Similarly, several complications of uncontrolled chronic hypertension have been reported in patients with 11*β*-OHD, including left ventricular hypertrophy, hypertensive retinopathy, hypertensive nephropathy, and ischemic heart disease [3, 17].

However, in our case, although there were no chronic complications aside from microalbuminuria, the patient experienced transient right hemiplegia due to severely uncontrolled blood pressure. This raised the possibility of bilateral adrenalectomy before changing the GC treatment; indeed, positive outcomes with complete discontinuation of antihypertensive agents have been reported in some cases of patients whom underwent bilateral adrenalectomy due to malignant hypertension [17–19].

In our case, after switching to DR-HC there was a significant improvement in ABP control, and a normal ABP profile was achieved while continuing therapy.

However, we do not observed an improvement of albuminuria but, even if microalbuminuria persisted in our patient, a complete remission of hypertension has been described, not only with respect to the condition itself but also in terms of associated end-organ damages, such as left ventricular hypertrophy or heart failure with adequate GC supplementation [20, 21]; however, we could not exclude that remission of end-organ damage is influenced by the age of onset, duration, and severity of ABP hypertension itself, so further data are required.

Lastly, there was also a notable positive effect on gonadal function, with improvement of A4/T ratio, suggesting an augmented contribute of testis in testosterone production [22], and also on fertility, with the restoration of a spermatogenesis. The improvement of the semen analysis was related to a reactivation of the hypothalamic–pituitary axis, as observed by the gradual increase of both FSH and LH levels due to a better control of androgen excess; however, it remained only partial since there was no improvement of TARTs related testicular involvement.

In fact, we did not observe any regression in adrenal or testicular lesions, which at least remained stable over time.

In conclusion, we reported a clinical case that encompassed all the known complications related to poor disease control in patients with CAH due to 11*β*-OHD, including significant adrenal enlargement, TARTs, infertility, and drug-resistant hypertension.

The management of this patient was further complicated by the presence of drug-resistant epilepsy with several drug interactions.

We further demonstrated the potential use of DR-HC for the first time in this clinical setting, where other medical options were not feasible or acceptable to the patient.

So, this case underscores the complexity of managing these patients, pinpointing the need for tailored GC therapy that considers each comorbidity and its respective treatments, with potential drug interactions.

Finally, our case emphasizes the importance of adequate and prompt disease control to limit long-term complications, which are often only partially reversible and could significantly impact fertility and CV health in these patients.

## 4. Conclusions

GCs are the cornerstone of treatment for CAH; however, both undertreatment and overtreatment can have equally harmful effects on these patients. In our case, hydrocortisone was replaced with dexamethasone to enhance compliance after puberty. Despite administering a substantial dose of dexamethasone, hormone levels remained poorly controlled due to drug interactions. As a result, the patient experienced all the complications associated with inadequate disease management. We describe the use and effectiveness of a relatively new formulation of DR-HC in this rare context. It is likely that new specific compounds could be even more effective for CAH types other than 21-OHD, but currently, no data are available.

## Figures and Tables

**Table 1:** Biochemical and hormonal exam results of the patient during various glucocorticoid treatment schedules.

Parameters	First evaluation	Dexamethasone 0.75 mg/day	6 months after DR-HC 20 mg/day	Last evaluation on DR-HC 25 mg/day	Normal values
- Renin, mIU/L	<2.0	<2.0	<2.0	8.4	4.4–46–1
- Aldosterone, pmol/L	11	26.9	50.5	71	70–1086
- Serum potassium, mmol/L	3.3	3.7	3.5	3.6	3.4–4.5
- Serum sodium, mmol/L	142	140	143	142	136–145
- ACTH, ng/L	1063	980	814	590.8	4.7–48.8
- 17-OH-progesterone, nmol/L	45.8	63	16.20	15.29	0.88–6.24
- Progesterone, nmol/L	5	5.01	5.5	4.9	0.16–0.47
- Androstenedione, nmol/L	111	157	88.5	75.8	1.7–12–2
- Total testosterone, nmol/L	17.85	7.24	12.91	17.20	9.72–38.17
- Androstenedione/total testosterone ratio	6.23	21.7	6.86	4.4	<0.5
- LH, U/L	2.3	<0.1	2.8	6.7	1.5–9.2
- FSH, U/L	3.4	<0.1	4.0	7.0	1.5–12.4

## Data Availability

The data supporting this case report are available within the main text and further details may be sourced from the original articles cited.
